# Thoracolumbar Cortical Screw Placement with Interbody Fusion: Technique and Considerations

**DOI:** 10.7759/cureus.1419

**Published:** 2017-07-02

**Authors:** Michael Karsy, Michael R Jensen, Kyril Cole, Jian Guan, Andrea Brock, Chad Cole

**Affiliations:** 1 Department of Neurosurgery, University of Utah; 2 Department of Neurosurgery, Stanford University

**Keywords:** cortical screws, thoracolumbar fixation, fusion, spine

## Abstract

A surge in interest in cortical bone trajectory (CBT), first described by Santoni in 2009, may be a result of its numerous advantages, including reduced surgical incision length and lateral dissection, limited disruption of the facet joints, and decreased blood loss. In addition, CBT offers improved screw pullout strength and the ability to perform hybrid constructs with pedicle screws using minimally invasive approaches. However, one of the main limitations of the technique involves the small screw size, which limits the potential for long-segment constructs. We describe a technique involving a more in-line anatomical trajectory, allowing for larger screw diameters. A feasibility study using a cadaveric model was performed and evaluated. Moreover, a focused review of the literature on the use of CBT was performed. Screw entry points are located along the inferomedial aspect of the facet and angled superolaterally. The use of this technique allows for the placement of larger screws (4.5 to 6.5 mm diameter) without pedicle breaches along with the alignment of screw heads from L1 to S1. In addition, the technique can be performed using stereotactic navigation or fluoroscopy. A direct, more in-line technique allows for larger screws to be placed using CBT. This technique can be combined with minimally invasive approaches. The potential advantages of the CBT technique support its use as a probable alternative to traditional pedicle screw fixation techniques.

## Introduction

First described in 2009 by Santoni et al., cortical bone trajectory (CBT) is a relatively new surgical approach used to reduce morbidity from spinal lumbar fixation and fusion [1]. As providers have become familiar with its benefits, there has been a dramatic increase in its use. The technique allows varied screw starting positions and trajectories. Initial reports indicate a 30% improved pullout strength compared with traditional pedicle screws, and increased insertional torque has been noted with screw placement [1]. Additional benefits include reduced incision length, reduced surgical dissection because the dissection of the transverse processes is not required, and reduced estimated blood loss (EBL) (Table 1) [2]. Biomechanical and finite element models suggest improved pullout strength and comparable fixation during multiaxis testing [3-5]. In addition, the technique may be important in osteoporotic patients who have poorer pedicle bone quality but retain cortical bone accessible by CBT [6]. However, one of the limitations of CBT remains the smaller screw sizes, compared with the pedicle screws, which must be used and that impacts the ability to use these screws on long-segment fusions or with deformities. We describe here a technique using a more in-line, anatomical approach to the pedicle that allows larger cortical screws.

**Table 1:** Focused review of biomechanical and clinical studies involving cortical screws Direct lateral interbody fusion (DLIF); transforaminal lateral interbody fusion (TLIF); computerized tomography (CT); pedicle screw TLIF (PS-TLIF); cortical screw TLIF (CS-TLIF); cortical screws with posterior lumbar interbody fusion (CS-PLIF)

Reference	Sample size of study	Biomechanical or clinical study type	Findings
Santoni et al. 2009 [1]	5	Biomechanical	First description of cortical screws A 29.00 ± 2.89 mm length and 4.66 ± 0.24 mm diameter cortical screw showed no difference in pullout strength compared to a pedicle screw (367.54 ± 23.65 vs. 287.59 ± 35.64, p=0.08) or toggle testing Three specimens were osteoporotic by a dual-energy X-ray absorptiometry scan
Perez-Orribo et al. 2013 [3]	28	Biomechanical	Combinations of cortical and pedicle screws with or without DLIF or TLIF devices were evaluated Pedicle screw-rod constructs were stiffer in axial rotation There was no difference in stability with DLIF and pedicle or cortical screw constructs TLIF-pedicle screw constructs were only stiffer with lateral bending
Matsukawa et al. 2013 [4]	100	Biomechanical	Morphometric vertebral (L1 to L5) analysis showing increased cortical screw length (36.8-38.3 mm) and little change in lateral (8.5-9.1^o^) or cephalad (25.5-26.2^o^) angles
Baluch et al. 2014 [7]	17	Biomechanical	Compared cortical and pedicle screws showing increased resistance to toggle testing (184 vs. 102 cycles, p=0.002) and increased force to displace screws (398 vs. 300N, p=0.004) No difference in pullout strength (1722 vs. 1741N, p=0.837)
Matsukawa et al. 2015 [8]	30	Biomechanical	Finite element model of pedicle (6.5 x 40 mm) and cortical (5.5 x 35 mm) screws Greater pullout strength for cortical screws (p=0.003) and increased stiffness with cephalocaudal force (p<0.05), mediolateral force (p=0.0001), and flexion/extension stiffness (p<0.05) Decreased lateral bending stiffness (p<0.05) and axial rotation stiffness (p<0.001)
Lee et al. 2015 [9]	79	Clinical	Randomized clinical trial of pedicle (n=39) vs. cortical (n=40) screws for degenerative spine disease, excluding osteoporotic patients No difference in 12-month fusion on dynamic X-rays (87.2% vs. 89.5%, p=0.81), CT scan (87.2% vs. 92.1%, p=0.61), visual analog scale, or Oswestry disability index scores Lower operating time, incision length, and estimated blood loss with cortical screws
Kasukawa et al. 2015 [10]	26	Clinical	Comparison of PS-TLIF and CS-TLIF procedures showing reduced estimated blood loss and operative time with CS-TLIF but equivalent fusion, lordosis, and screw positioning
Kojima et al. 2015 [11]	222	Biomechanical	CBTs evaluated for vertebral bodies showing significant variance in bone density compared to pedicle screw trajectories
Matsukama et al. 2015 [12]	30	Biomechanical	Finite element modeling of osteoporotic L4 vertebrae performed with a comparison of the cortical (5.5 x 35 mm), pedicle (7.5 x 40 mm), and combined hybrid screw approaches Significantly increased fixation in flexion (268%), extension (269%), lateral bending (210%), and axial rotation (178%) seen for hybrid screws compared to cortical screws alone (p<0.1)
Mai et al. 2016 [13]	180	Biomechanical	Increased bone mineral density for cortical screw trajectories than for pedicle screws across patient ages and if patients show overall osteoporosis
Chin et al. 2016 [14]	60	Clinical	A total of 30 patients with cortical screws in an outpatient setting matched to in-hospital pedicle screws Significant improvement in visual analog scale score (p=0.001) and Oswestry disability index (p=0.004) seen for cortical screws Similar fusion rates at two years
Matsukama et al. 2016 [15]	202	Clinical	Factors correlating with facet joint violation included age>70 years, vertebral slip>10%, and adjacent facet joint degeneration
Matsukama et al. 2016 [16]	50	Biomechanical	Thoracic cortical screws evaluated with a starting point at the intersection of the lateral two-thirds of the superior articular process and the inferior border of the transverse process Cranial targeting toward the posterior one-third of the superior endplate Higher average insertional torque is seen for cortical compared to pedicle (1.02 ± 0.25 vs. 0.66 ± 0.15 Nm, p<0.01) screws
Matsukama et al. 2016 [17]	20	Biomechanical	Finite element modeling evaluating cortical screw biomechanics Larger screw diameter (4.5-6.5 mm) impacted a pullout strength greater than pedicle screws Longer screws (25-40 mm) increased pullout strength and axial fixation Percentage screw length within the vertebral body was more important than the actual screw length
Sakaura et al. 2016 [2]	95	Clinical	CS-PLIF compared to historical control Significantly greater Japanese Orthopedic Associated Score (JOA) 13.7 to 23.3 vs. 14.4 vs. 22.7, p<0.05) and lower adjacent-segment disease (3.2 vs. 11.0%, p<0.05) with cortical screws
Sakaura et al. 2016 [18]	193	Clinical	Significantly higher caudal screw loosening with lumbosacral CS-PLIF compared to floating CS-PLIF (46.2 vs. 6.0%)

## Technical report

Indications and Contraindications

The indications for using CBT are similar to those for using traditional transpedicular screws (Table 2). These include spinal stenosis, spondylolisthesis, or degenerative spondylosis with motion (movement of more than 3 mm seen on flexion-extension x-rays). Use in patients with coronal deformities and having a Cobb angle of more than 30°, where correction is desired, is relatively contraindicated. The considerable forces necessary for axial derotation maneuvers are likely best applied with traditional pedicle screws. In addition, significant sagittal imbalance and the use of longer constructs (more than three levels) may be more amenable to traditional pedicle screw techniques. Multilevel scoliosis or kyphosis requiring multiple osteotomies remains a relative contraindication. Pars defects, whether congenital or traumatic, or absent cortical bone for screw purchase, are also absolute contraindications for CBT.

**Table 2:** Indications and contraindications for CBT screws

Indications	Contraindications
One- and two-level posterior spinal fusion for unstable spondylolisthesis or spondylosis with more than 3-mm movement	Derotation during coronal or sagittal deformity
Minimally invasive one-level hybrid (cortical-pedicle) screw constructs	Multilevel scoliosis
Salvage procedures for failed pedicle screw placement	Multilevel kyphosis
	Congenital or traumatic pars defects
	Absent cortical bone for screw purchase

Preoperative Preparation

Standard preoperative preparation can be performed similarly to the workup for traditional pedicle screws. An evaluation of computerized tomography (CT) scans to assess bone anatomy and quality, magnetic resonance imaging (MRI) to evaluate the ligamentous integrity and thecal sac compression, and upright X-rays to visualize dynamic instability and spinopelvic parameters can be performed. Preoperative anesthetic preparation is standard, although the expected reduction in operative time and EBL may be considerations in patients with a higher American Society of Anesthesia (ASA) physical status classification, where pedicle screws may not be possible. Similarly, the use of cortical screws for patients with osteoporosis or adjacent segment disease can be specifically considered.

Equipment Needs

**Figure 1:**
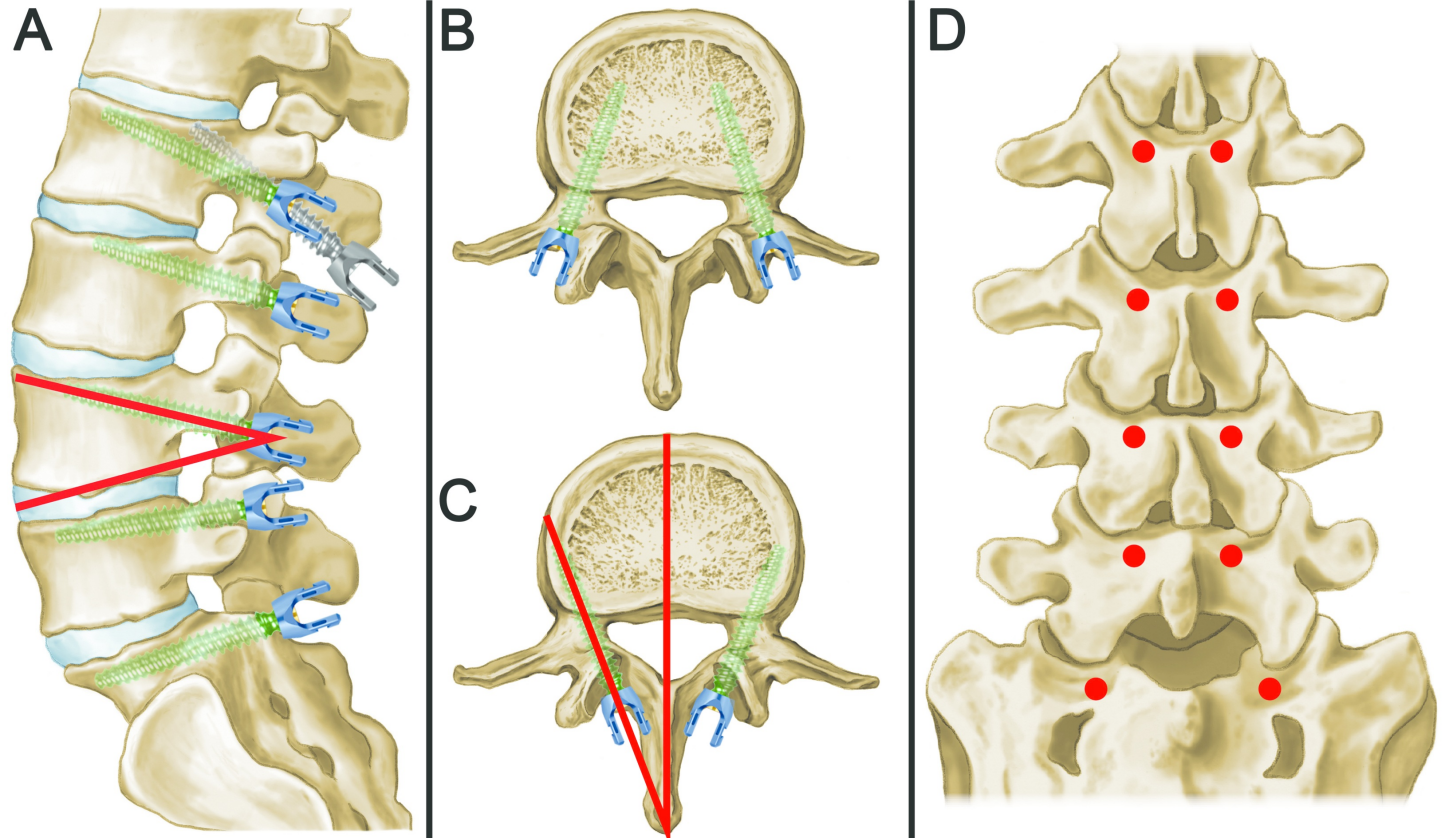
Schematic of CBT (A) Sagittal schematic view with a modified cortical bone trajectory (CBT) showing cephalad screw angulation. Cephalad angles found on 14° for L1 and L2, 16° for L3 and L4, 12° for L5, and 9° for S1 (shown, for example, with a red angle at L4 between the screw and the inferior endplate). A typical cortical screw trajectory is shown in comparison (gray) to our modification (green) at the L1 level. (B) Traditional pedicle screw trajectories are shown with the medialization of screws and entry at the intersection of the transverse process and the lateral edge of the pars interarticularis. (C) An axial CBT is shown with the lateralization of screws. Lateralized angles of 6-­7° for L1, 7-­8° for L2, 10-­11° for L3, 12­-13° for L4, 14-­15° for L5, and 15° for S1 (shown, for example, with a red angle). (D) Coronal view showing the screw entry sites around the inferomedial aspect of the facet (red dots). Entry positions align to allow rod placement without the need for offset screw heads.

Multiple manufacturers are available to provide cortical screw instrumentation. In this study, K2M (Leesburg, VA) was utilized for the cadaveric and case studies (Figure 1). Because multiple cortices of bone are engaged with CBT, the pedicle screw tract must be prepared with a power drill. It is recommended that a high-speed 3.0-mm cutting or coarse diamond drill bit be used to open the posterior cortex for the entry point. Subsequently, a small, hand-held drill with a 3.0-mm drill bit should be used along with a drill guide to cannulate the pedicle across the three different cortical surfaces along the desired screw trajectory. It is recommended that a navigation system is used to guide CBT, especially when learning the technique. C-arm fluoroscopy can be used, but only with a solid understanding of the trajectory and bony landmarks.

Technique

**Figure 2:**
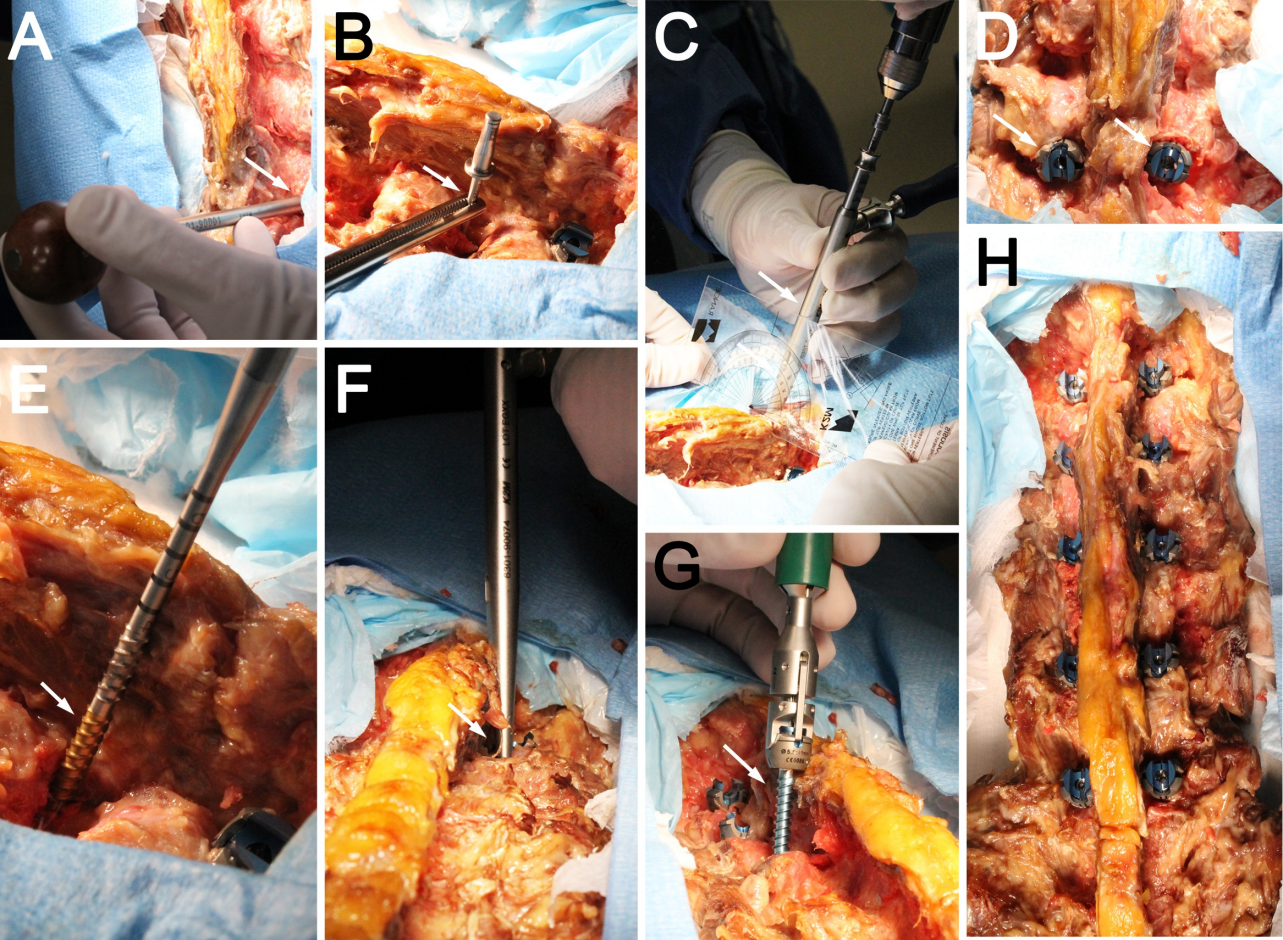
Photographs showing cadaveric screw placement using CBT (A) An awl or drill is used to create a pilot hole at the inferomedial aspect of the facet (arrow). (B) A marker can be left in position with an axial fluoroscopy view to ensure the proper screw starting position (arrow). (C) A 1-mm undersized tap is used to generate a trajectory (arrow). Note the cephalad angulation of the guide. The drilled trajectory is probed to ensure no breaches. (D) Completed cortical screws showing the placement of the screw heads at the inferomedial aspect of the facet (arrow). (E) A self-guiding tap is shown with length markers (arrow). (F) A completed tap highlights the lateralization required for the screw (arrow). The tapped hole is measured to evaluate the needed screw length. (G) A cortical screw is shown during placement (arrow). (H) Completed cortical screws from L1 to L5 and aligned screw heads are shown (arrow).

**Figure 3:**
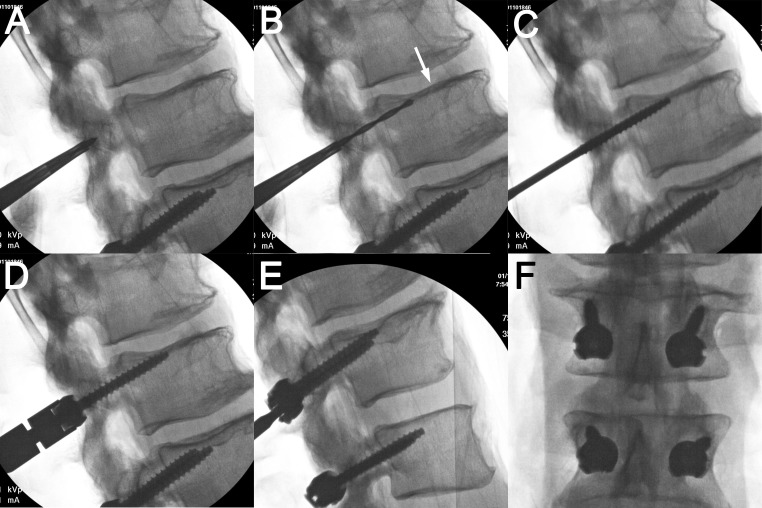
Representative cadaver radiographs Lateral fluoroscopic images showing the placement of the pilot hole via an awl (A), the drilled trajectory aiming toward the midpoint of the superior endplate (arrow) (B), the tapped trajectory (C), and the final cortical screw placement with close approximation of the screw head and facet joint without a superior endplate breach (D). Representative (E) lateral and (F) anteroposterior x-ray films of instrumented cadaveric vertebrae from L3 to L4 are shown.

The patient is placed prone on a standard spine frame with imaging/navigation capabilities using standard positioning, preparation, and draping (Figures 2-3). A midline incision is used. The superior/inferior margins are defined by the superior endplate of the upper instrumented vertebrae and the inferior endplate of the lower instrumented vertebrae. For a single-level procedure, the incision is approximately 35-40 mm. Sharp dissection is carried out in standard fashion of the medial borders of the facet joints and the lateral pars interarticularis.

The entry point is defined by the medial border of the superior articular process of the vertebra to be instrumented and the location on the pars interarticularis approximately 3­5 mm inferior to the inferior border of the facet joint complex. This is generally approximated by the inferior margin of the transverse process (not directly visualized). A clock face can be imagined around the facet with the left pedicle entry at the 5 o’clock position and the right pedicle at the 7 o’clock position.

The high-speed burr is now used to open the cortex to a depth of approximately 2 mm. The drill with the 3.0-mm drill bit is then used to develop the screw tract. The drill is directed from the entry point approximately 25° cephalad and 8° laterally. Visualizing the clock face again, this is approximately toward the 10 o’clock and 2 o’clock positions for the left and right screws, respectively.

A tap that is 1.0 mm smaller than the intended screw is used to develop threads for the full length of the screw. The tap is advanced to a depth of at least 30 mm. Care is taken to remain within the bony borders. The desired endpoint is defined by the lateral border of the superior endplate, anterior to the midpoint of the vertebral body when viewed from a lateral position. Specialized taps that disengage can be left within the screw tract and used to distract the disc space for discectomy and preparation for an interbody fusion device. Alternatively, a marker (k-wire or Steinman pin, Skylar Instruments, West Chester, PA) can be placed in each screw tract to demarcate the tract while performing laminectomies and/or disc space preparation. The spinous processes can be removed before tapping and screw placement if they inhibit the technique.

CBT can potentially be applied safely from any lower thoracic level [16] to the first sacral level. The use of CBT in the sacrum is unique because of the lack of a true cortical pedicle. The 5 o’clock position of the pedicle is still used as an entry point, and the tract is developed toward the ala of the sacrum with a lateral angle of approximately 15 degrees. Bicortical screws are recommended for better purchase while remaining cognizant of the L5 nerve root. S1 level cortical screws can possibly be placed using 7.5 x 35-40 mm screws. The use of this trajectory in combination with CBT at L5 will provide good screw alignment for the introduction of rods. The drill trajectory for the L5 screw is generally approximated as perpendicular to the floor, with a 10-degree lateral angulation in most patients. This typically allows for a 6 x 35 mm screw to be placed. The remaining lumbar levels all have similar trajectories, as described in the technique. If pelvic fixation is desired, the use of traditional S2-alar-iliac (S2AI) constructs may be complementary to the CBT technique with a low construct profile. However, further studies involving the biomechanical stability and outcomes for L5/S1 constructs or attachment to S2AI instrumentation in conjunction with CBT are required.

Laminectomy and Interbody Fusion

The screws are not placed until the final stages of the posterior procedure, as the surgeon’s access to the canal and disc space may be impeded by the limited exposure and the screw head. This may be a potential disadvantage of cortical screws compared to pedicle screws.

Any posterior decompression or interbody fusion procedures may be completed at this point. Care should be taken to preserve the region of the pars for the CBT screw, to avoid weakening the bone and creating the risk of screw fracture. Particular attention should be paid to the removal of the inferior articulating process. Care should be taken to limit potential fractures or the extension of the bone removal, which would encroach into the cannulated pars interarticularis-pedicle junction. Mindful dissection of the medial-lateral aspect of the lamina-pars interarticularis junction is also needed. Maintaining the cortical surface surrounding the cannulated pars interarticularis/pedicle will maintain the greater strength of the larger screw. After the posterior decompression and interbody grafts have been placed, the appropriate screws are placed with the screw inserted to the depth of the thread. Overtightening of the screw, resulting in strain on the cortical bone as well as the impingement of the screw head, causing an inability to reposition, should be avoided.

Thought should be given to the choice of interbody fusion devices to be used. If placed from a posterior approach, transforaminal devices can create a challenge because of the limited lateral exposure used for CBT. This may require the removal of the medial two-thirds of the inferior and superior articulating processes. Oblique anterior and paramedian devices may also be used.

The decortication of the remaining posterior arch and the disruption of the internal facet joints of the levels to be fused are completed. If a wide facetectomy is required during the decompression, the decortication of the lateral aspect of the superior articulating process along with the medial aspect of the upper instrumented vertebrae provides a less invasive approach and provides for robust fusion. Rods are placed and screws locked. Bone graft is applied, and the wound is closed in the appropriate fashion.

## Discussion

CBT Position

**Figure 4:**
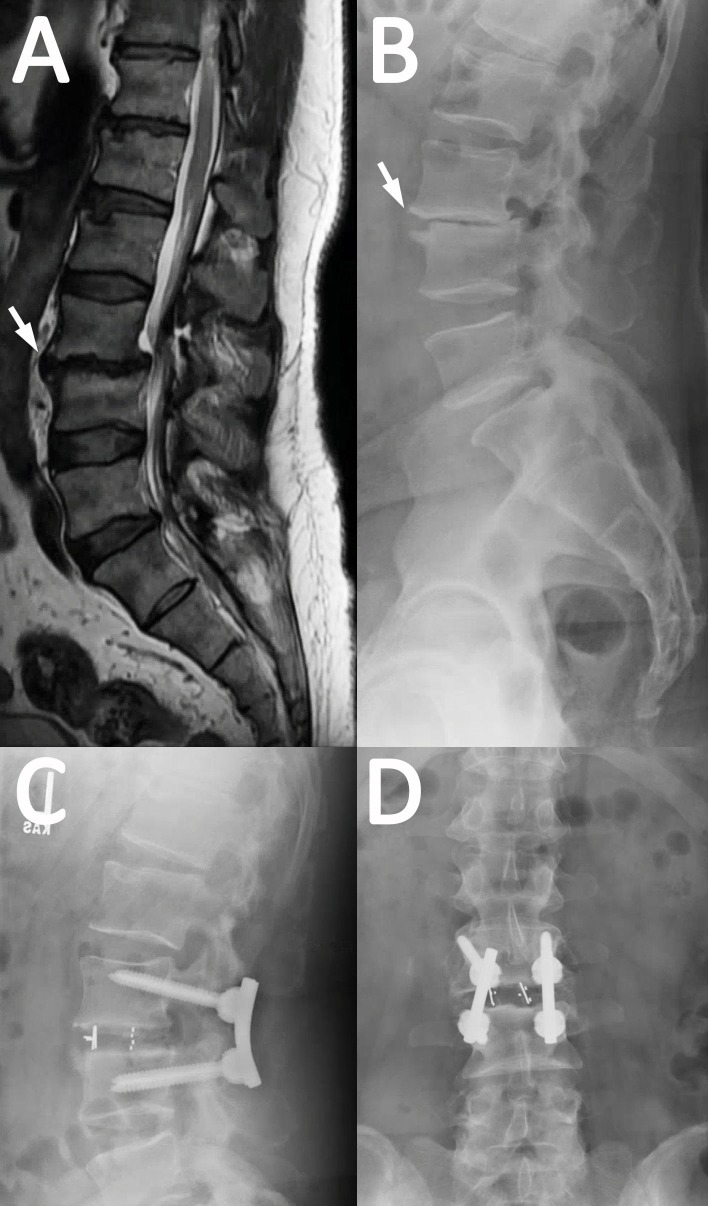
Representative case example using CBT A 58-year-old male presents with acute progression of chronic back pain with radiculopathy and subjective lower extremity weakness. He underwent an L3 laminectomy, L3/4 transforaminal lumbar interbody fusion (TLIF), and posterior spinal fusion. (A) Preoperative magnetic resonance imaging and (B) x-rays show spondylolisthesis of L3 on 4 and spinal stenosis (arrow). (C) Lateral and (D) anteroposterior x-rays after L3/4 TLIF using the cortical bone technique (CBT) are shown.

In contrast to the traditional pedicle screw that starts at the junction of the transverse process and the lateral pars interarticularis, the CBT screw begins at the medial position of the pars. Its trajectory is angled superolaterally compared with medialized pedicle screws. Prior studies have suggested lateral angles of 8­9° and cephalad angles of 25° [4-5]. An analysis of previous cadaveric and clinical data suggests the lateralized angles of 6-­7° for L1, 7­-8° for L2, 10­-11° for L3, 12-­13° for L4, 14-­15° for L5, and 15° for S1 (results not shown). Cephalad angles were 14° for L1 and L2, 16° for L3 and L4, 12° for L5, and 9° for S1 (results not shown). These results suggest that alterations in angle are important for screw positioning and can be performed using stereotactic screws or fluoroscopy. Such trajectories allow for the cannulation of the cortical surfaces at the posterior surface, within the pedicle and the annular ring of the vertebral body (Figure 4).

Advantages and Disadvantages of CBT Screws

Several distinct advantages of the CBT screw exist over traditional pedicle screws (Table 1). The trajectory of CBT can potentially avoid the thecal sac by aiming laterally, and it avoids the exiting nerve root by aiming superiorly. Reduced surgical incision lengths, dissection, and EBL may be possible but can depend on the surgeon and technique. This advantage can be useful in obese patients where soft tissue constraints add to the difficulty of using pedicle screws. The reduced disruption of the facet for screw placement may also reduce postoperative pain related to facet capsule and medial branch nerve injury [4]. One increased biomechanical advantage in osteoporotic patients involves cortical bone purchase resulting in reduced screw failure rates when evaluated computationally and via cadavers [3,6]. CBT may also be used as a salvage or reinforcing technique for traditional fusion constructs. The use of CBT can also avoid the use of polymethylmethacrylate and bone cement. Furthermore, the use of hybrid constructs for pedicle and cortical screws, such as quad screw constructs or combination pedicle-cortical screws, can be valuable strategies for greater fixation and more minimally invasive approaches.

Disadvantages can include a difficulty in performing decompression and interbody fusion with limited dissection or obstructing screw heads. Distinct techniques from pedicle screws, such as placing screws after decompression, may need to be employed. There is also a potential for misaligned screw heads requiring the use of off-set connectors, especially during the placement of hybrid constructs linking cortical and pedicle screws. Finally, the use of CBT screws for long-segment fusion constructs remains to be further explored. Biomechanical data shows improved pullout strength and multiaxial toggling for CBT; however, these results may not necessarily relate to patients or account for patient-specific factors.

## Conclusions

The use of a more in-line, anatomical approach may allow for larger-size cortical screws expanding the range of lumbar fusion possibilities and hybrid constructs. Here, techniques for such an approach are presented along with a focused review of the literature. Cephalad and lateralized angles are provided for potential use at any position on the lumbar spine. The advantages of CBT involve reduced surgical dissection, lower blood loss, and potential combinations using hybrid techniques with pedicle screws. Further study regarding the efficacy of this technique is required in patients in order to establish whether the modification of the technique can truly improve patient outcomes.
